# Advancing agricultural research using machine learning algorithms

**DOI:** 10.1038/s41598-021-97380-7

**Published:** 2021-09-09

**Authors:** Spyridon Mourtzinis, Paul D. Esker, James E. Specht, Shawn P. Conley

**Affiliations:** 1 Agstat Consulting, Athens, Greece; 2 Department of Plant Pathology and Environmental Microbiology, Pennsylvania State University, State College, PA 16801 USA; 3 Department of Agronomy and Horticulture, University of Nebraska-Lincoln, Lincoln, NE 68583-0915 USA; 4 Department of Agronomy, University of Wisconsin-Madison, Madison, WI 53706 USA

**Keywords:** Agroecology, Agroecology

## Abstract

Rising global population and climate change realities dictate that agricultural productivity must be accelerated. Results from current traditional research approaches are difficult to extrapolate to all possible fields because they are dependent on specific soil types, weather conditions, and background management combinations that are not applicable nor translatable to all farms. A method that accurately evaluates the effectiveness of infinite cropping system interactions (involving multiple management practices) to increase maize and soybean yield across the US does not exist. Here, we utilize extensive databases and artificial intelligence algorithms and show that complex interactions, which cannot be evaluated in replicated trials, are associated with large crop yield variability and thus, potential for substantial yield increases. Our approach can accelerate agricultural research, identify sustainable practices, and help overcome future food demands.

## Introduction

Increasing food demand will challenge the agricultural sector globally over the next decades^1^. A sustainable solution to this challenge is to increase crop yield without massive cropland area expansion. This can be achieved by identifying and adopting best management practices. To do so requires a more detailed understanding of how crop yield is impacted by climate change^2,3^ and growing-season weather variability^4^. Even with that knowledge, prediction is challenging because various factors interact with each other. For example, variability in soil type can interact with weather conditions and mitigate or aggravate climate-related impacts on crop yield^5,6^. Additionally, seed genetics (G) and crop management decisions (M), interact with the effect of environment (E: soil and in-season weather conditions), thereby resulting in a near infinite number of combinations of G × E × M that can impact crop yield.

Substantial variability in crop yield arises from the wide range of optimal to sub-optimal management observed in soybean farmers’ fields^7,8^. Reducing the frequency of lowest *vs*. highest yields has been proposed as an effective means to increase food production in existing crop land^9^. In that regard, replicated field experiments have been used to identify best management practices for several decades. Most commonly, the effectiveness of up to three management factors and their interactions are evaluated in a single location due to practical constraints (e.g., cost, logistics). By holding the background management constant, causal relationships are identified, and the effectiveness of the examined management practice/s is assessed. It is assumed that background management practices are optimal or at least relevant to what most farmers use in the region, which in fact may not be realistic for many farmers.

Multi-year-site performance trials that account for large environmental and background management variability is another common practice in agricultural research. Such trials usually estimate an average effect across environments and background cropping systems. Inevitably, the measured yield response magnitude and sign may not apply to all farms in the examined region. Other research approaches involve analysis of producer self-reported data^7,8^, which can capture yield trends attributable to producer management choice across large regions, but such studies lack sufficient power relative to establishing causality and evaluating complex high-order G × E × M interactions.

Process-based models have been extensively used to evaluate the effect of weather^10^ and management^11,12^ on crop yield. However, to obtain accurate estimates, the models require extensive calibration, which is not a trivial task due to the large number of parameters. Specifically, it has been shown that management is an important source of uncertainty in process-based models, which can lead to substantial and varying degree of bias in yield estimates across the US, even when using harmonized parameters^13^.

Given all the well-known deficiencies of current agricultural research methods, we argue that a method that allows environment-specific identification of unique cropping systems with the greatest yield potential is essential to meet future food demand. Here, by utilizing maize and soybean yield and management data from publicly available performance tests, plus associated weather data, and by leveraging the power of machine learning (ML) algorithms, we developed a method that can evaluate myriads of potential crop management systems and thereby identify those with the greatest yield potential in specific environments across the US.

## Results and discussion

**Figure 1:**
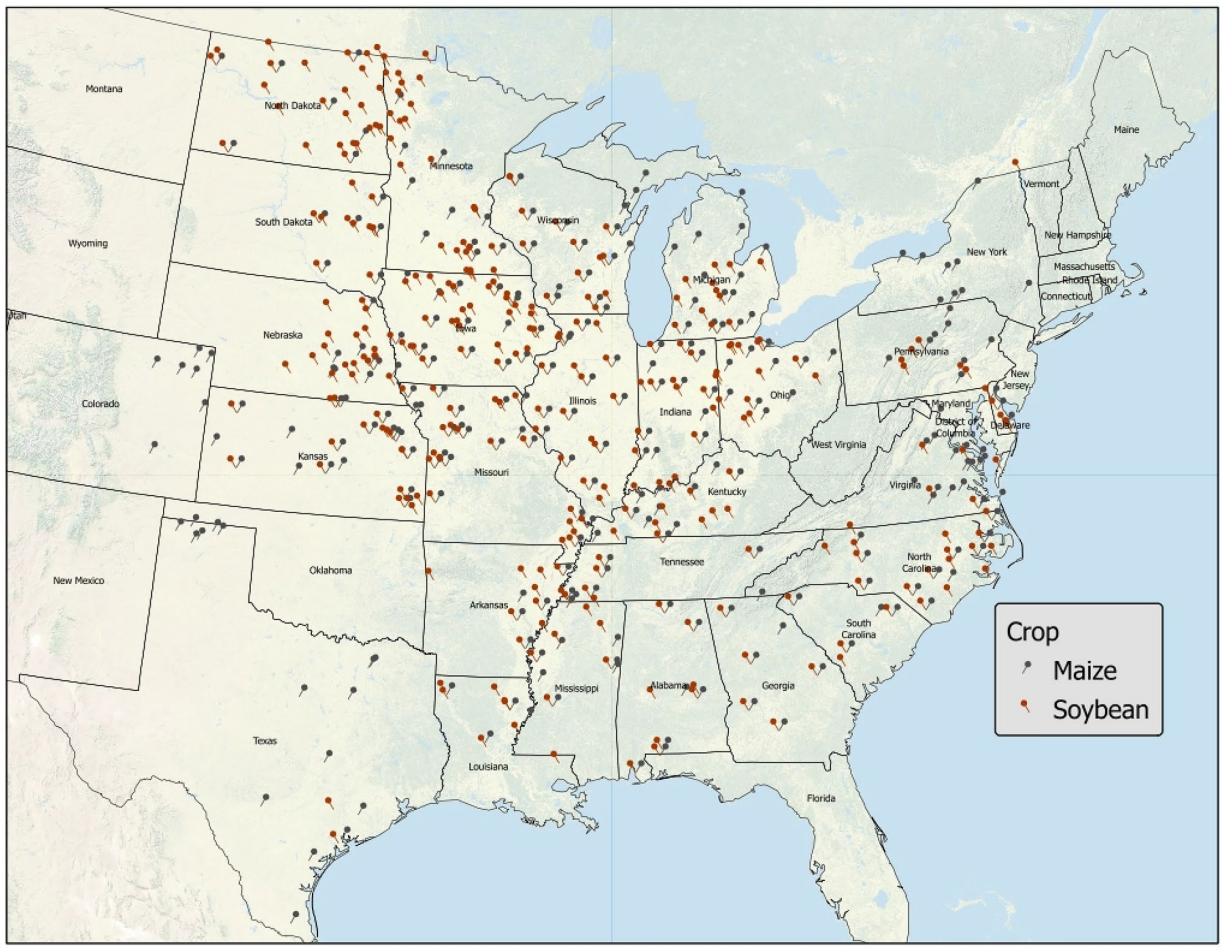
Locations where maize and soybean trials were performed during the examined period. The map was developed in ArcGIS Pro 2.8.0 (https://www.esri.com).

Two databases including yield, management, and weather data for maize (n = 17,013) and soybean (n = 24,848) involving US crop performance trials conducted in 28 states between 2016 to 2018 for maize and between 2014 to 2018 for soybean, were developed (Fig. 1). Crop yield and management data were obtained from publicly available variety performance trials which are typically performed yearly in several locations across each state (*see methods for more information*). Final databases were separated in training (80% of database) and testing (20% of database) datasets using stratified sampling by year, use of irrigation, and soil type. For each crop, an extreme gradient boosting (XGBoost, *see methods for more information*) algorithm to estimate yield based on soil type and weather conditions (E), seed traits (G) and management practices (M) was developed (see variables listed in Tables S1 and S2 for maize and soybean, respectively, and data science workflow in Fig. S1).

**Figure 2:**
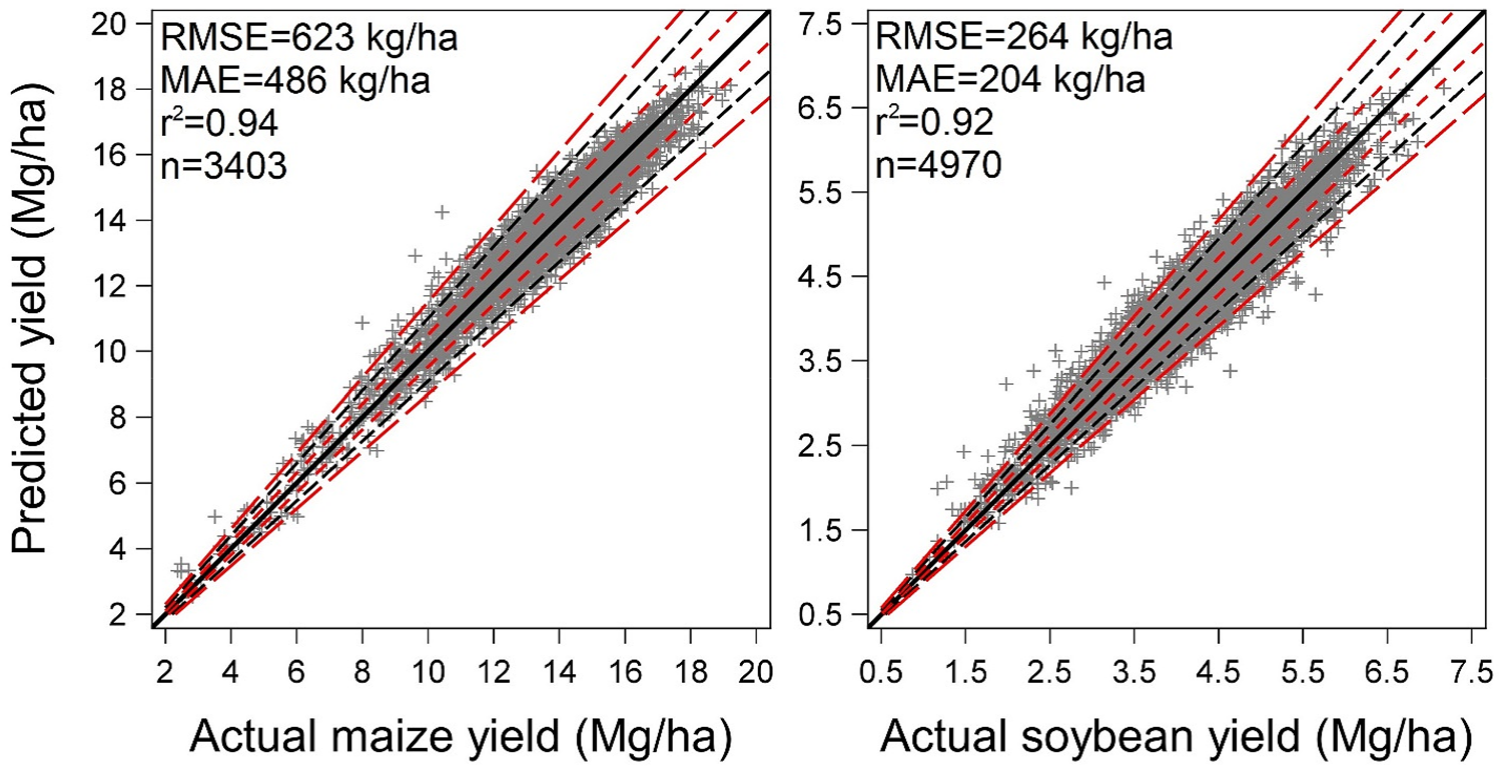
Actual versus algorithm-derived maize (left) and soybean (right) yield in test datasets. Black solid line indicates y = x, red short-dashed lines, black dashed lines, and red long-dashed lines indicate ± 5, 10, and 15% deviation from the y = x line. RMSE, root mean square error; MAE, mean absolute error; r^2^, coefficient of determination; n = number of observations. Each observation corresponds to a yield of an individual cropping system in a specific environment (location-year).

The developed algorithms exhibited a high degree of accuracy when estimating yield in independent datasets (test dataset not used for model calibration) (Fig. 2). For maize, the root mean square error (RMSE) and mean absolute error (MAE) was a respective 4.7 and 3.6% of the dataset average yield (13,340 kg/ha). For soybean, the respective RMSE and MAE was 6.4 and 4.9% of the dataset average yield (4153 kg/ha). As is evident in the graphs (Fig. 2), estimated yields exhibited a high degree of correlation with actual yields for both algorithms in the independent datasets. For maize and soybean, 72.3 and 60% of cases in the test dataset deviated less than 5% from actual yields, respectively. Maximum deviation for maize and soybean reached 43 and 70%, respectively. Data points with deviations greater than 15% from actual yield were 1.5% in maize and 3.6% in soybean databases. These results suggest that the developed algorithms can accurately estimate maize and soybean yields utilizing database-generated information involving reported environmental, seed genetic, and crop management variables.

**Figure 3:**
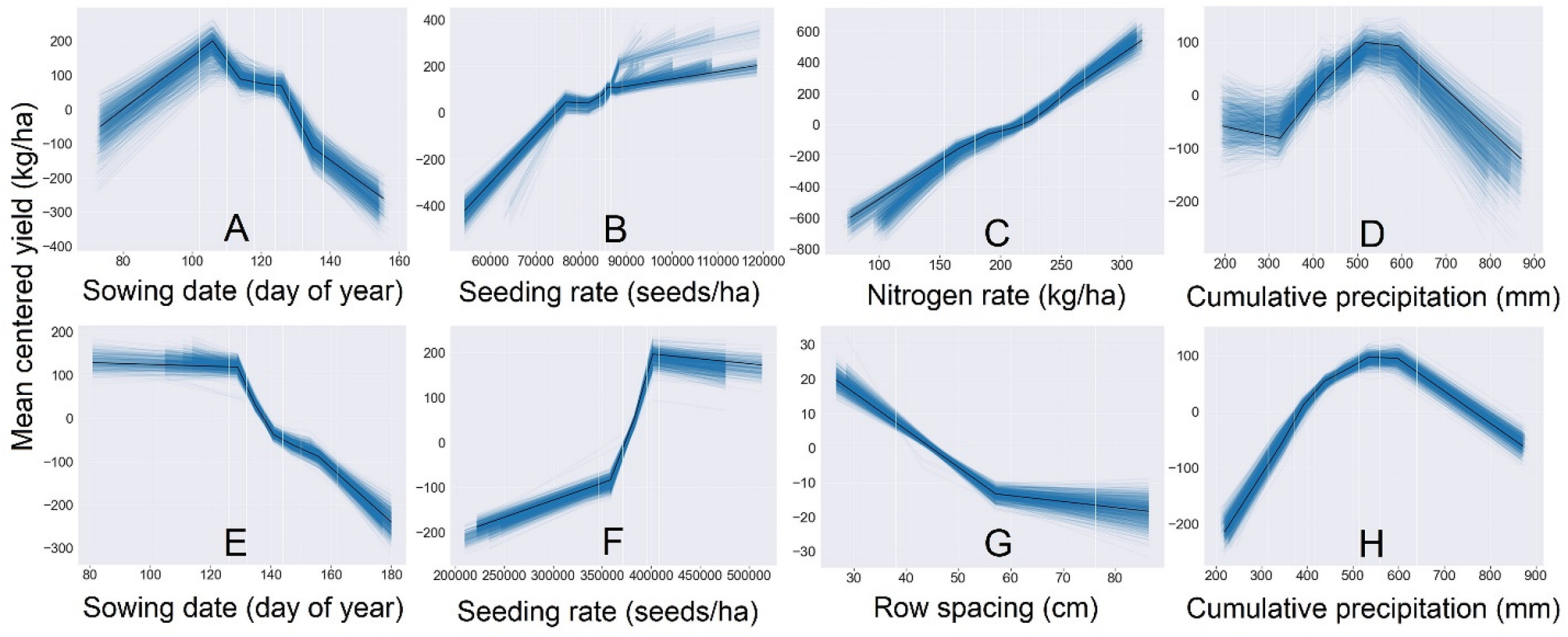
Accumulated local effect plots for maize sowing date (**A**), seeding rate (**B**), Nitrogen fertilizer rate (**C**), and cumulative precipitation between June and September (mm) (**D**), and soybean sowing date (**E**), seeding rate (**F**), row spacing (**G**), and cumulative precipitation between June and September (mm) (**H**).

In contrast to statistical models, ML algorithms can be complex, and the effect of single independent variables may not obvious. However, accumulated local effects (ALE) plots^14^ can aid the understanding and visualization of important and possibly correlated features in ML algorithms. For both crops, indicatively important variables included sowing date, seeding rate, nitrogen fertilizer (for maize), row spacing (for soybean) and June to September cumulative precipitation (Fig. 3). Across the entire region and for both crops, the algorithm-derived trends suggest that above average yields occur in late April to early May sowing dates, but sharply decrease thereafter. Similar responses have been observed in many regional studies across the US for both, maize^15–18^ and soybean^19^. Similarly, simulated yield curves due to increasing seeding rate are in close agreement with previous maize^20,21^ and soybean^22^ studies. The maize algorithm has captured the increasing yield due to increasing N fertilizer rate. The soybean algorithm suggests that narrower row spacing resulted in above average yield compared to wider spacing. Such response has been observed in many regions across the US^23^. Season cumulative precipitation between 400 and 700 mm resulted in above average yields for both crops.

**Figure 4:**
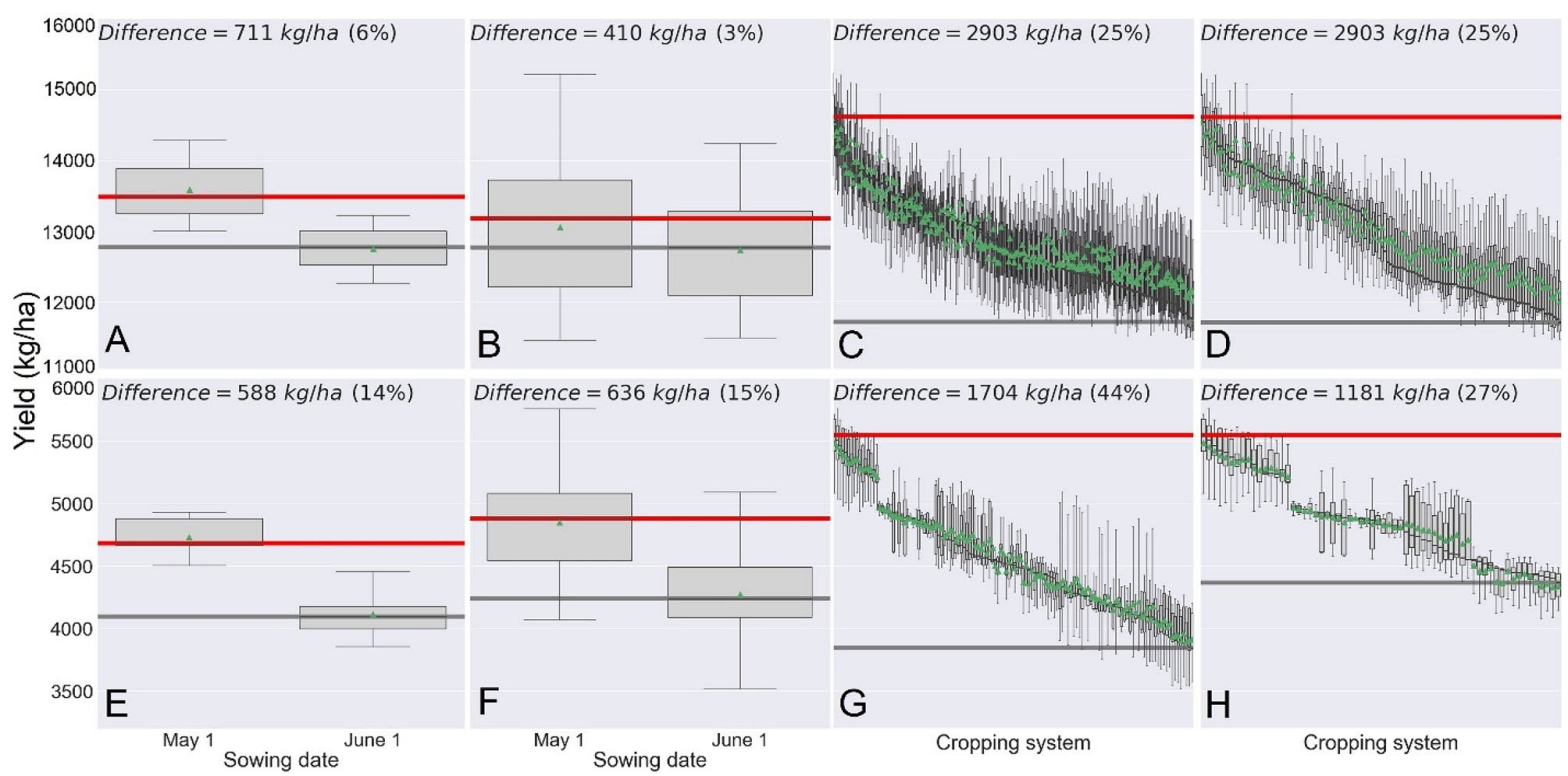
Maize yield difference (in kg/ha and percentage) due to sowing date (May 1st vs. June 1st) for a single identical background cropping system (**A**), maize yield difference due to sowing date when averaged across 256 (3 years × 256 cropping systems = 768 year-specific yields) (**B**), maize yield variability in each of the 256 cropping systems (**C**), and maize yield variability in each of the 128 cropping systems with early sowing (**D**). Soybean yield difference due to sowing date (May 1st vs June 1st) for a single identical background cropping system (**E**), soybean yield difference due to sowing date when averaged across 128 (5 years × 128 cropping systems = 640 year-specific yields) (**F**), soybean yield in each of the 128 cropping systems (**G**) and soybean yield variability due in each of the 64 cropping systems with early sowing (**H**). Within each panel, the horizontal red and grey lines indicate the boxplot with maximum and minimum yield, respectively. In the left four panels, boxes delimit first and third quartiles; solid lines inside boxes indicate median and green triangles indicate means. Upper and lower whiskers extend to maximum and minimum yields. Each maize and soybean cropping system is a respective 8-way and a 7-way interaction of management practices in a randomly chosen field in Wisconsin, USA (Table S3 and S5, respectively).

The responses in the ALE plots (Fig. 3) suggest that these algorithms have captured the general expected average responses for important single features. Nevertheless, our databases include hundreds of locations with diverse environments across the US and site-specific crop responses which may vary due to components of the G × E × M interaction. We argue that, instead of examining a single or low-order management interactions, site-specific evaluation of complex high order interactions (a.k.a. cropping systems) can reveal yield differences that current research approaches cannot fully explore and quantify. For example, sowing date exerts a well-known impact on maize and soybean yield. For each crop separately, by creating a hypothetical cropping system (a single combination of all management and traits in Tables S1 and S2) in a randomly chosen field in south central Wisconsin (latitude = 43.34, longitude = -89.38), and by applying the developed algorithms, we can generate estimates of maize and soybean yield. For that specific field and cropping system (out of the vast number of management combinations a farmer can choose from), maize yield with May 1st sowing was 711 kg/ha greater (6% increase) than June sowing (Fig. 4A). By creating scenarios with 256 background cropping system choices (Table S3), the resultant algorithm-derived yield estimate difference for the same sowing date contrast (averaged across varying cropping systems) was smaller but still positive (3% increase), although the range of possible yield differences was wider (Fig. 4B). However, when comparing, instead of averaging, the estimated yield potential among the simulated cropping systems, a 2903 kg/ha yield difference (25% difference) was observed (Fig. 4C). Interestingly, when focusing on the early sown fields that were expected to exhibit the greatest yield, the same yield difference was observed (Fig. 4D). This result shows that sub-optimal background management can mitigate the beneficial effect of early sowing (Table S4).

In the case of soybean, a May 1st sowing resulted in greater yield (588 kg/ha; a 14% increase) than a June 1st in the single background cropping system (Fig. 4E). The result was consistent when yield differences due to sowing date were averaged across 128 background cropping system choices (Table S5) (Fig. 4F). Similar to what was observed in maize, among all cropping systems, yield varied by 1704 kg/ha (44% difference) (Fig. 4G). When focusing only on the early sown fields, a 1181 kg/ha yield difference (27% yield increase) was observed (Fig. 4H). In agreement with maize, this result highlights the importance of accounting for sub-optimal background management which can mitigate the beneficial effect of early sowing (Table S6).

We note here the ability of farmers to change management practices can be limited due to an equipment constraint (e.g., change planter unit row width) or simply impossible (e.g., change the previous year’s crop). Thus, recommended management practices that were evaluated in studies that used specific background management may not be applicable in some instances. The benefits of the foregoing approach, which involves extensive up-to-date agronomic datasets and high-level computational programing, can have important and immediate implications in future agricultural trials. Our approach allows for more precise examination of complex management interactions in specific environments (soil type and growing season weather) across the US (region covered in Fig. 1). The ability to extract single management practice information (even across cropping systems) is also possible by utilizing ALE plots, or by calculation of the frequency at which a given level/rate of a management practice appeared among the highest yielding cropping systems (Tables S4 and S6).

Among all available 30-d weather variables, many were strongly correlated in both crop databases (Figs. S2 and S3 for maize and soybean, respectively). Models using all 30-d interval variables with r < 0.7 (Tables S8 and S9) showed minimal to no performance gain compared to the final more parsimonious models that included season-long weather variables (Fig. S4). Thus, we consider the length of periods we chose to represent well the approximate successive 60-d pre-sowing, 120-d in-season, and 60-d post-harvest segments of growing season in the US (Fig. S7). Season-long weather conditions have been used in previous studies^13,24^, and it has been shown that choice of growing season does not affect climate-related effects on crop yield^25,26^.

As an additional sensitivity analysis, we developed ALE plots for the algorithms using the aforementioned 30-d weather variables (Fig. S8). For major management practices, there were no differences in simulated responses between the algorithms that used multiple 30-d weather variables and the final chosen algorithms that used longer intervals (Fig. 3). Repeating the analysis for the same hypothetical cropping system in the same Wisconsin location using the algorithms developed with the 30-d weather conditions, the observed trends were consistent with the season-long weather algorithms, although the simulated yields were numerical different (Fig. S9). Nevertheless, across all representations of weather conditions (algorithms with 30-d intervals and season-long), the levels/rates of management practices in the 5% highest and lowest yielding maize and 5% highest soybean cropping systems with early sowing date were identical, apart from manure use in maize. Based on these results, we consider the algorithm-derived yield estimates robust to different representations of seasonal weather variability.

It appears that several different cropping systems can result in similar high yield for both crops (Fig. 4C,D,G,H). This is in agreement with other agricultural decision maker tools^27^. Moreover, it is common for neighboring farms to attain similar crop yield despite the use of a different cropping system, suggesting that a single optimal solution does not necessarily exist and that different combinations of management practices, when they interact with environment, can still result in similar high yields. Since the effect of environment is ever-changing, the high level of complexity of synergies between G × E × M suggests that long-term optimization of single management factor may not be possible^28^, which further highlights the importance of accounting for the effect of the entire cropping system at the field level.

The approach we present here should not be considered as a crop yield forecasting exercise. There have been several attempts to forecast crop yields using deep neural network methods (e.g.,^29,30^). In contrast, the algorithms we present here can generate hypothetical experimental data that can be used to rapidly examine G × E × M interaction for both maize and soybean across the US. Of the millions of possible G × E × M combinations, our ML algorithms can identify hidden complex patterns between G × E × M combinations for yield optimization that may be non-obvious, but once identified, worthy of field test confirmation. Farmers can use the algorithms to gain insights about optimum management interactions in their location-specific environment (known soil type × expected weather conditions), and to identify farm factors that may be too costly to alter without a priori reason (generated by the model) for doing so. Researchers can compare expected yield across thousands of hypothetical cropping systems and use the results as a guide to design more efficient future field-based crop management practice evaluation experiments.

We note that this approach should not be considered as a substitute of replicated trials. To the contrary, replicated field trials performed by Universities are continually needed to serve as an excellent source of high-quality unbiased data which can be used to train even more comprehensive algorithms. The major issue with current performance trial data is that a great amount of management information is not reported. Usually, only information relevant to the examined management factors in each trial are reported, which inevitably results in missing values (Tables S1 and S2), or even in absence of important variables (e.g., number and dates of split fertilizer application). As we have highlighted here, the high order and complex background management interactions should not be considered as irrelevant.

## Conclusions

Agricultural experiments repeated every year in hundreds of locations across the US generate a vast amount of crop yield and management datasets which are useful for broad inferences (average effect of a management practice across a range of environments). Such datasets have, to date, remained disconnected from each other, and are difficult to combine, standardize, and properly analyze. In the presented work, we overcame these issues by developing large databases and by leveraging the power of ML algorithms. We argue that our algorithms can advance agricultural research and aid in revealing a currently hidden yield potential in each individual farm across the US.

## Methods

Crop yield and management data were obtained from publicly available variety performance trials which are typically performed yearly in several locations across each state^31^. Recorded, trial-specific, management practices for maize included use of irrigation, tillage practice, seeding rate, row spacing, sowing date, previous crop, fertilizer (N, P, and K), use of manure, cultivar’s maturity, insecticide traits and use of seed treatments (Table S1). For soybean, use of irrigation, foliar fungicide, tillage practice, seeding rate, row spacing, sowing date, previous crop, and cultivar maturity were recorded (Table S2).

Since data were collected from different states and years, it was assumed that reported management practices (general categories) were consistent across all locations. Additionally, the type and application method of fertilizer was rarely reported. Similarly, there was a lack of information on the active ingredient and rates of seed treatments and foliar applied products. We acknowledge that this lack of information, as we state in the discussion section, is a limitation of our databases and our assumption, that the way different management practices are reported across different states is consistent, may have contributed to the observed unexplained variability.

For both databases, data entry was performed manually. Additionally, for both crops, soil type was recorded and weather data (Table S7) were retrieved from the DAYMET^32^ database for each year and set of coordinates. DAYMET daily data are reasonably accurate when means or totals are calculated over extended periods^33^. Therefore, means and sums for three periods (90–150, 151–270, and 271–330 days of year) (Tables S1 and S2) and 30-d periods (Tables S8 and S9) were calculated. The different sets of weather variables were used in different models to assess their impact in model accuracy.

The exact coordinates for each site were not reported in the trial reports. Therefore, approximate coordinates, based on the nearest reported city, were used for each unreported site. When unmanageable production adversities were reported (e.g., hail, damage due to deer etc.), the associated data were not used. Missing values were present in almost all management-related variables in both databases (Tables S1 and S2). Since the data were derived from designed experiments, levels of management were not a result of response to external factors (e.g., weather conditions) but were researcher’s decisions to answer specific research questions (e.g., crop yield response to different sowing dates or maturity ratings), no missing data imputation was performed.

The first step before data analysis was to examine correlations among the weather variables. Due to their strong collinearity (Figs. S3 and S4 for maize and soybean, respectively), only those with Pearson r < 0.7 were retained for subsequent analyses. The final maize database included seven weather variables (Table S1) and the final soybean database included eight weather variables (Table S2). Categorical variables were one-hot encoded and then databases were separated in training (80% of database) and testing (20% of database) datasets. To ensure adequate representation of growing environments in both, the training and testing portions of the data, stratified sampling was performed by year, use of irrigation, and soil type. For each crop, an extreme gradient boosting (XGBoost) algorithm^34^ was trained to predict final yield as a response of the aforementioned weather and management variables listed in Tables S1 and S2. The hyperparameters were optimized using the training dataset and included number of estimators, tree depth, number of leaves, minimum sum of instance weight in node, learning rate, subsample percentage, column sample by tree and by level, gamma, alpha and lambda parameters. To efficiently tune the hypermeters, Bayesian optimization was performed using “hyperopt” in Python 3.6.9 with tenfold cross validation. The combination of the hypermeters that resulted in the lowest root mean square error (RMSE) in the tenfold cross validations was chosen as the final model which was further evaluated on the test portion of the data (Fig. 2 in main document).

Accumulated local effects (ALE) plots^14^, which are robust to correlation among independent variables, were developed for indicative and important variables using 1000 Monte Carlo simulations. These plots are useful to visualize how individual features influence the predictions of the developed “black-box” algorithms. To perform the evaluation for the “what if” scenarios, the final algorithms were applied on hypothetical cropping systems in a randomly chosen field in south central Wisconsin (latitude = 43.34, longitude =  − 89.38) and weather conditions in 2016–2018 for maize and 2014–2018 for soybean. Boxplots were used to visually evaluate the results.

## Supplementary Information

Supplementary Information.

## Data Availability

The datasets generated during and/or analyzed during the current study are available from the corresponding author on reasonable request.
