# Supplementary material for: Advancing agricultural research using machine learning algorithms
Source: Sci Rep. 2021 Sep 9;11:17879. doi: 10.1038/s41598-021-97380-7 (PMC8429560; doi:10.1038/s41598-021-97380-7)
Supplement: Supplementary file 1 — Supplementary Information. [file 41598_2021_97380_MOESM1_ESM.docx]

**Title**: Advancing agricultural research using machine learning algorithms.

**Authors:** Spyridon Mourtzinis^1*^, Paul D. Esker^2^, James E. Specht^3^, Shawn P. Conley^4^.

**Affiliations:**

^1^Agstat Consulting, Athens, Greece.

^2^Department of Plant Pathology and Environmental Microbiology, Pennsylvania State University, State College, PA, 16801, United States.

^3^Department of Agronomy and Horticulture, University of Nebraska-Lincoln, Lincoln, NE, 68583-0915, United States.

^4^Department of Agronomy, University of Wisconsin-Madison, Madison, WI, 53706, United States.

*Correspondence to: [agstat001@gmail.com](mailto:agstat001@gmail.com)


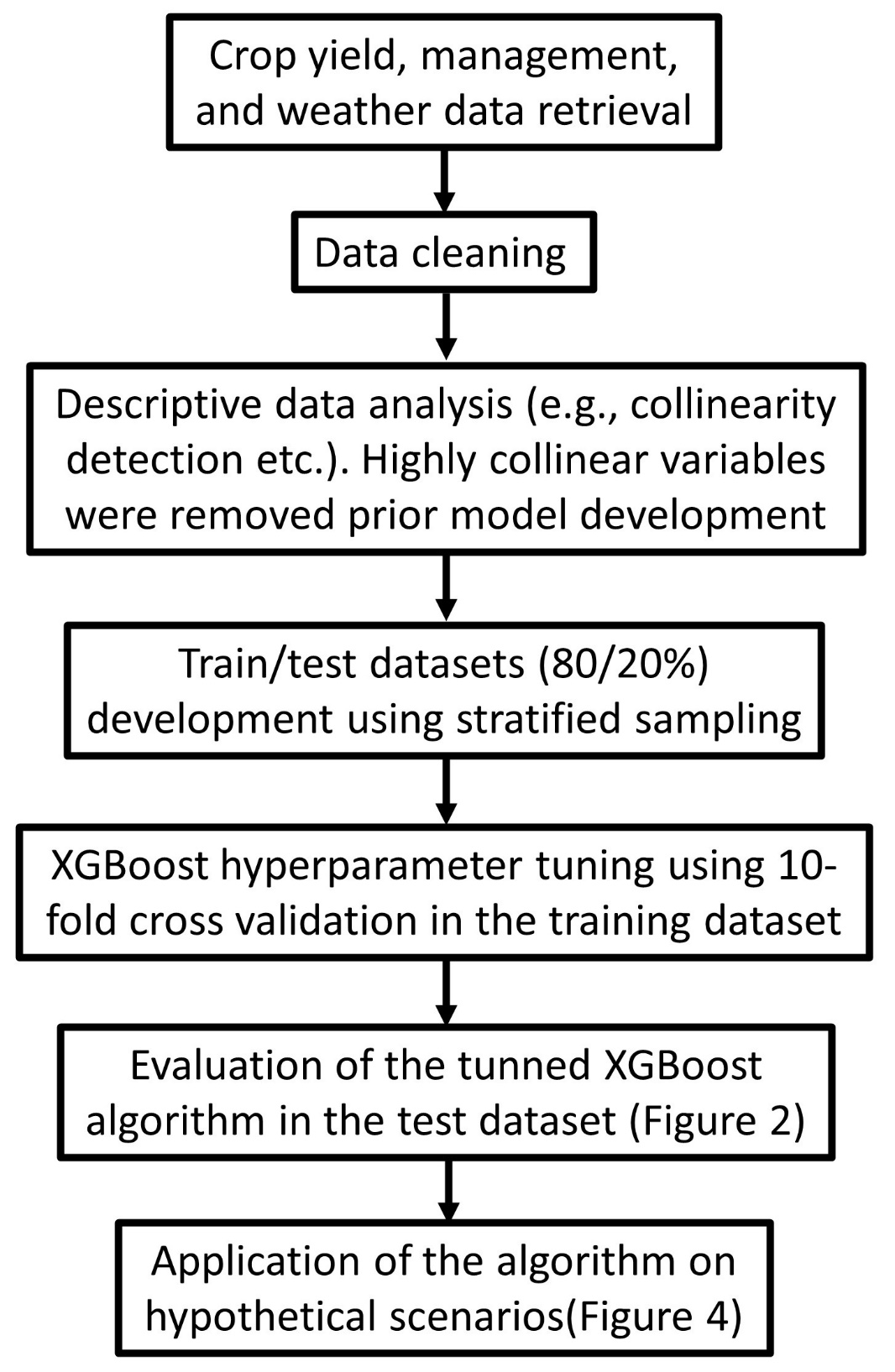


**Fig. S1**. Data science workflow.


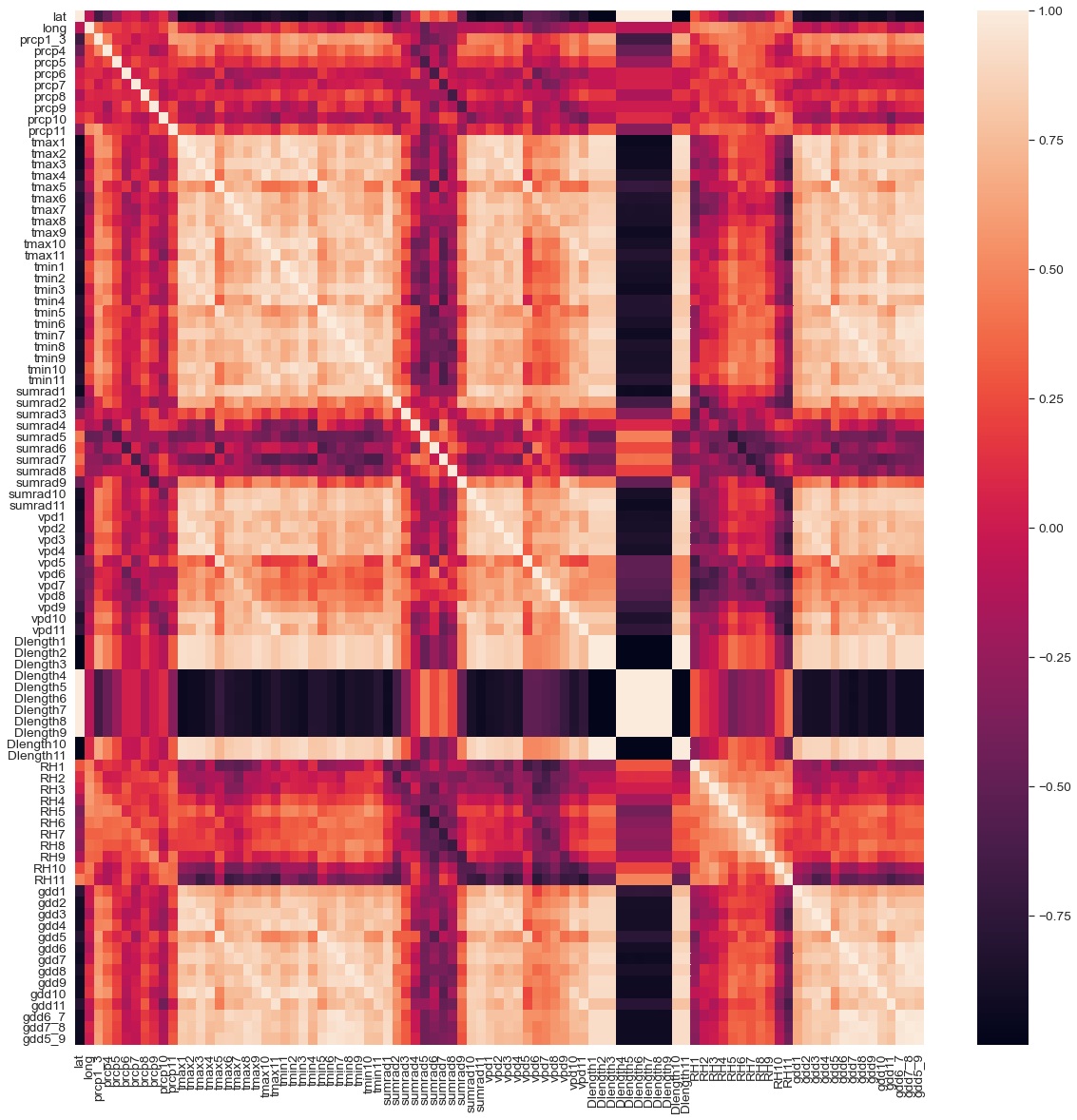


**Fig. S2**. Correlation of 30-d weather variables in maize database. Weather variables include cumulative precipitations (prcp), maximum and minimum air temperature (tmax and tmin, respectively), solar radiation (sumrad), vapor pressure deficit (vpd), daylength (Dlength), relative humidity (RH) and growing degree days (gdd). The numbers next to each variable show the 30-d interval (e.g., tmax1 is the maximum temperature during the first 30 days of the year etc.). The color gradient of the bar at the right of the figure shows the strength and direction of the correlation between variables.


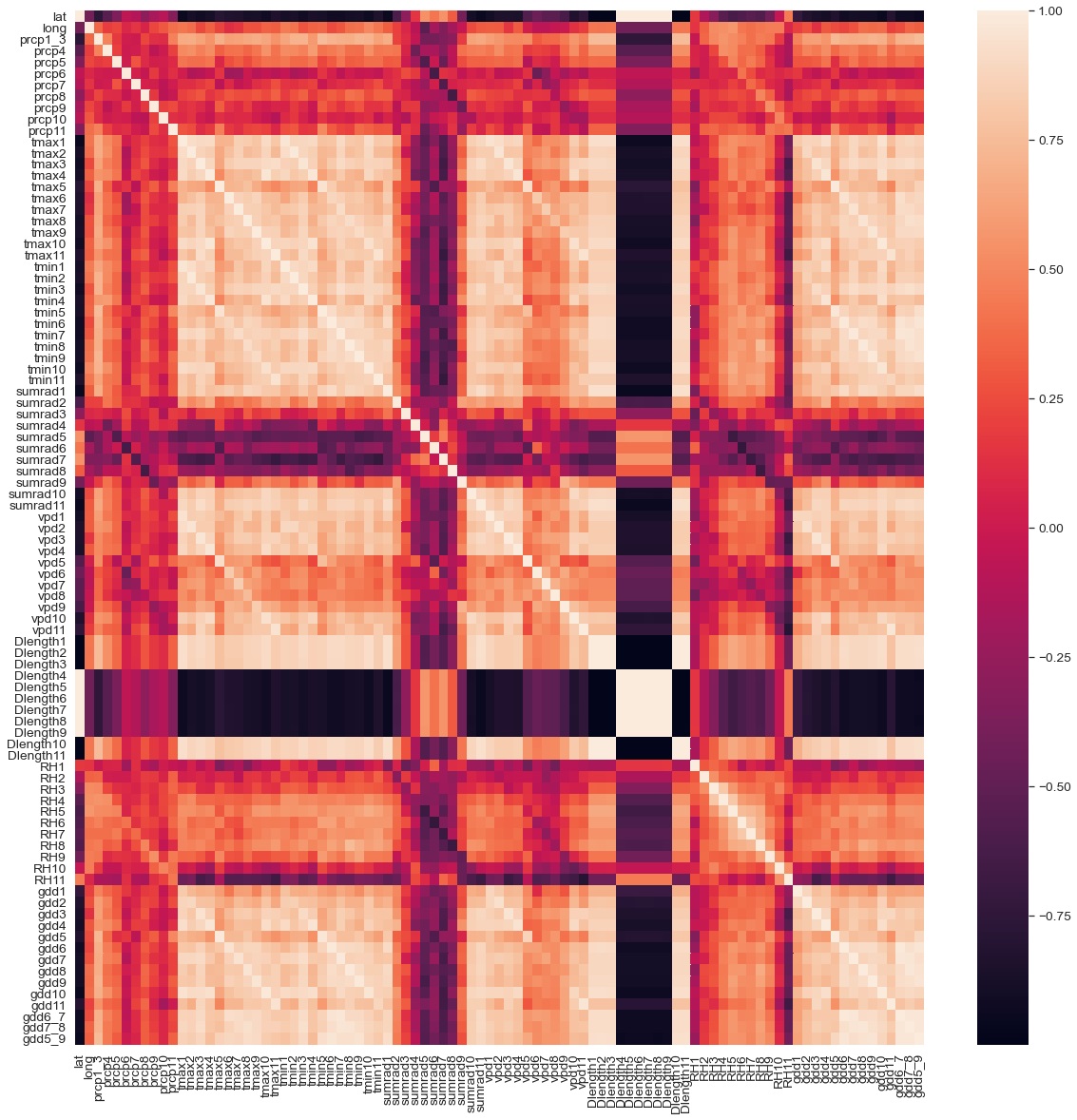


**Fig. S3**. Correlation of 30-d weather variables in soybean database. Weather variables include cumulative precipitations (prcp), maximum and minimum air temperature (tmax and tmin, respectively), solar radiation (sumrad), vapor pressure deficit (vpd), daylength (Dlength), relative humidity (RH) and growing degree days (gdd). The numbers next to each variable show the 30-d interval (e.g., tmax1 is the maximum temperature during the first 30 days of the year etc.). The color gradient of the bar at the right of the figure shows the strength and direction of the correlation between variables.


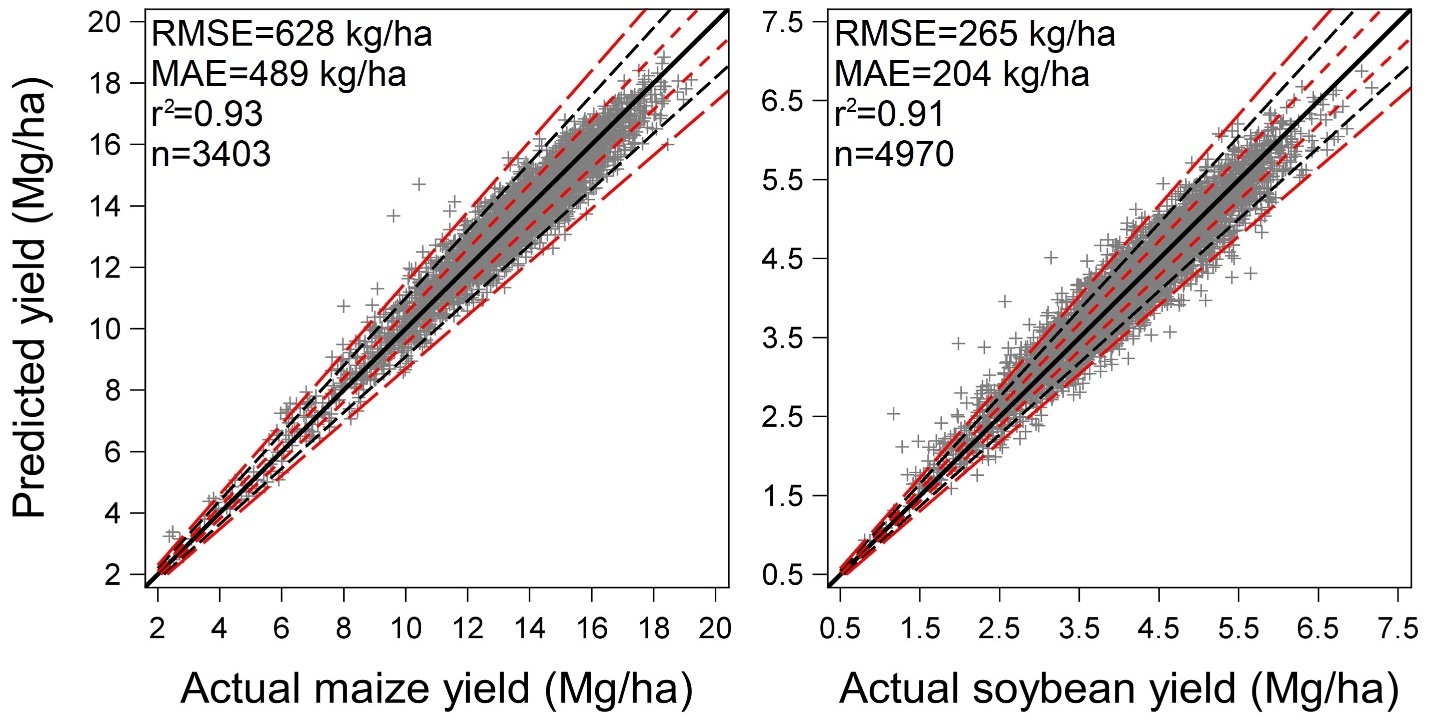


**Fig. S4**. Actual *vs* algorithm-derived yield in the testing portion of the for maize (left) and soybean (right) datasets, and when using 30-d weather variables with r < 0.7 (see **Table S4** and **S5**). Black solid line indicates y=x, red short-dashed lines, black dashed lines, and red long-dashed lines indicate ± 5, 10, and 15% deviation from the y=x line. RMSE, root mean square error; MAE, mean absolute error; r^2^, coefficient of determination; n=number of observations. Each observation corresponds to a yield of an individual cropping system in a specific environment (location-year).


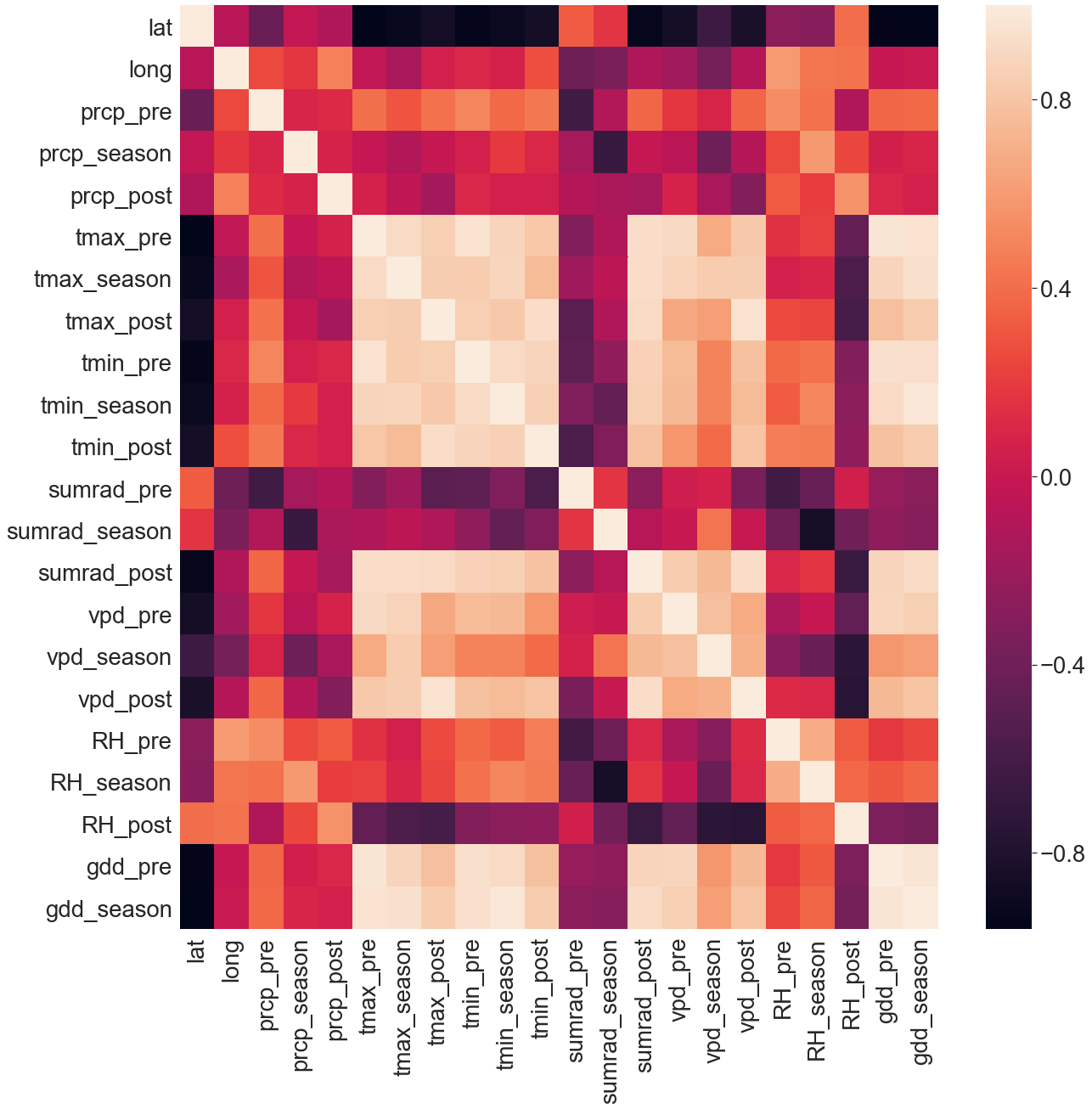


**Fig. S5**. Correlation of weather variables in maize database. Weather variables include cumulative precipitations (prcp), maximum and minimum air temperature (tmax and tmin, respectively), solar radiation (sumrad), vapor pressure deficit (vpd), relative humidity (RH) and growing degree days (gdd). For each variable, sums and means for three periods (pre: 90-150, season: 151-270, and post: 271-330 days of year) were calculated. The color gradient of the bar at the right of the figure shows the strength and direction of the correlation between variables.


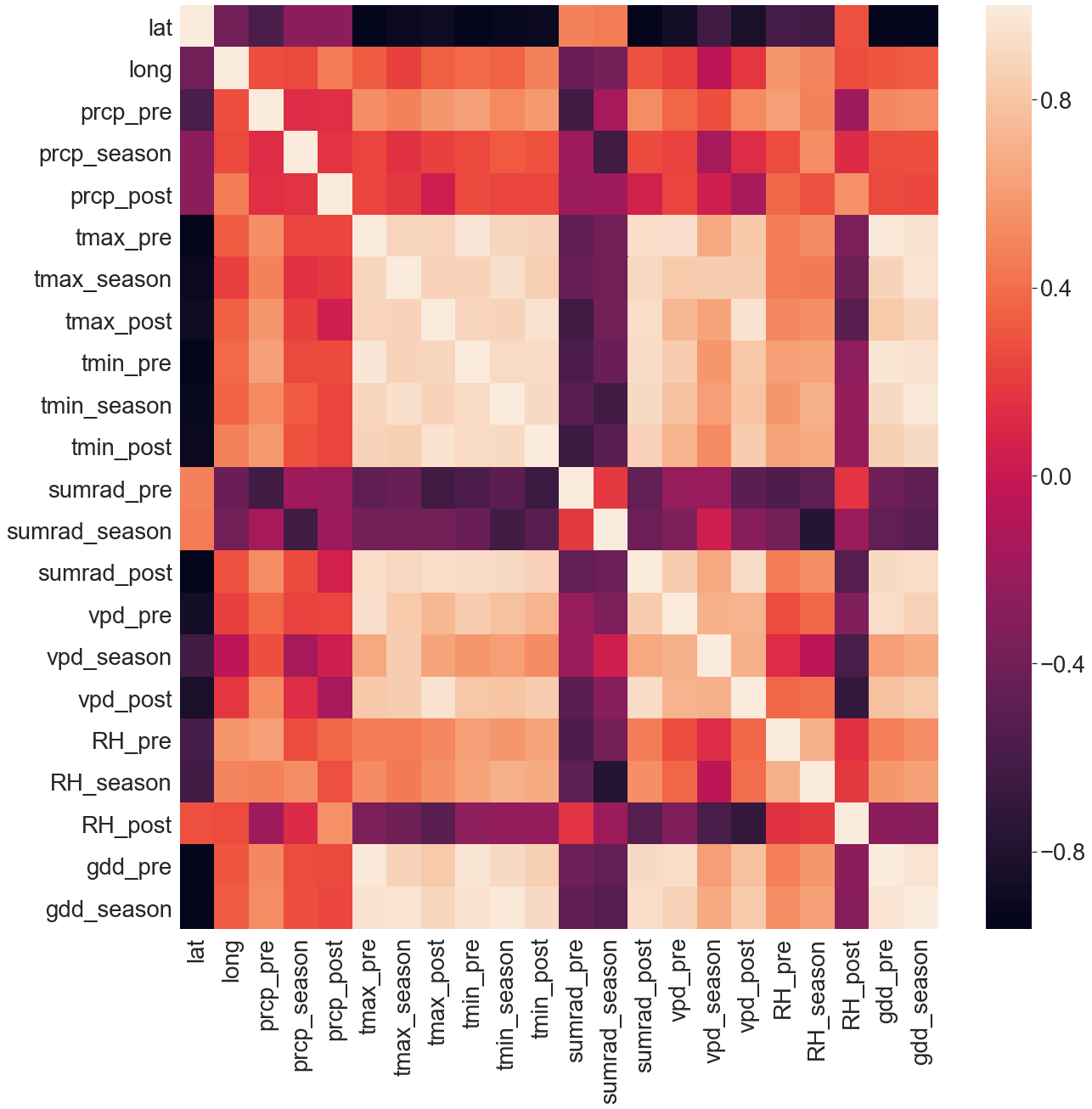


**Fig. S6**. Correlation of weather variables in soybean database. Weather variables include cumulative precipitations (prcp), maximum and minimum air temperature (tmax and tmin, respectively), solar radiation (sumrad), vapor pressure deficit (vpd), relative humidity (RH) and growing degree days (gdd). For each variable, sums and means for three periods (pre: 90-150, season: 151-270, and post: 271-330 days of year) were calculated. The color gradient of the bar at the right of the figure shows the strength and direction of the correlation between variables.


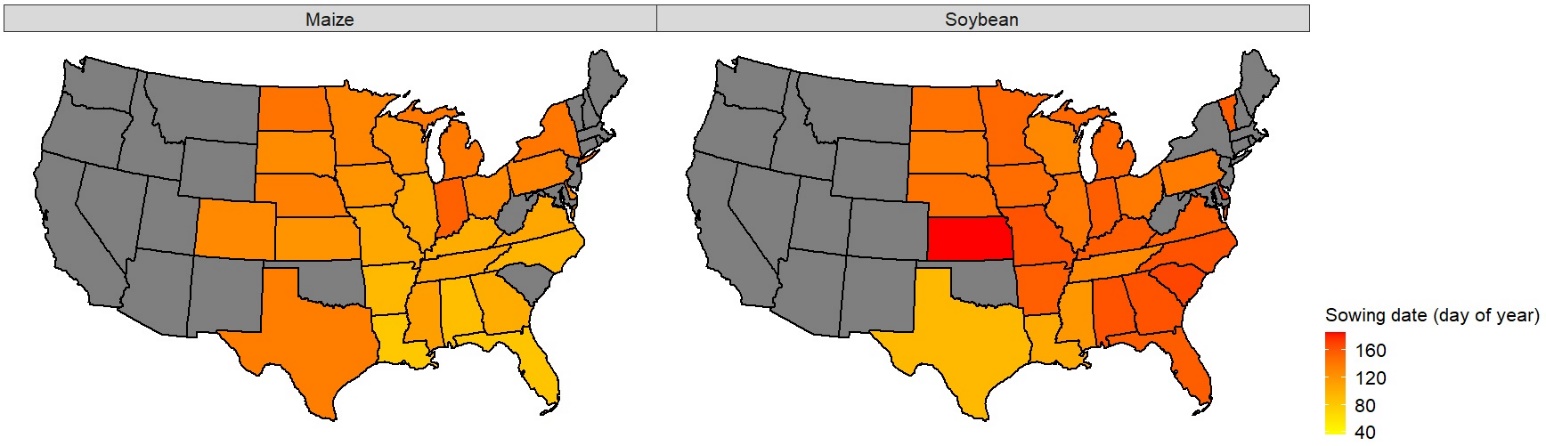


**Figure S7.** Average sowing dates in maize (left) and soybean (right) US-wide databases. Data from individual locations and years were grouped within states. The figure was developed in R 4.0.3 (https://www.R-project.org/)


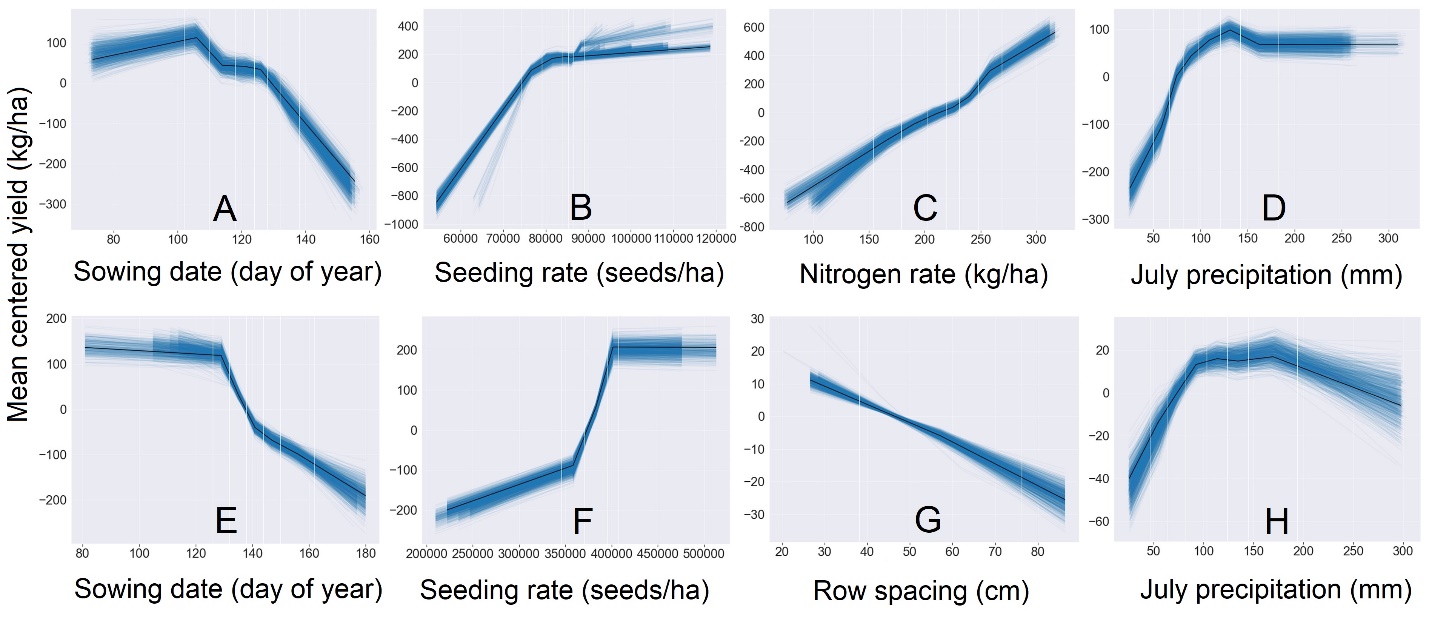


**Figure S8.** Accumulated local effect plots when using 30-d weather variables (**Table S4** and **S5** for maize and soybean respectively) for sowing date (A), seeding rate (B), Nitrogen fertilizer rate (C), and July precipitation (D) in maize, and sowing date (E), seeding rate (F), row spacing (G), and July precipitation (H) in soybean.


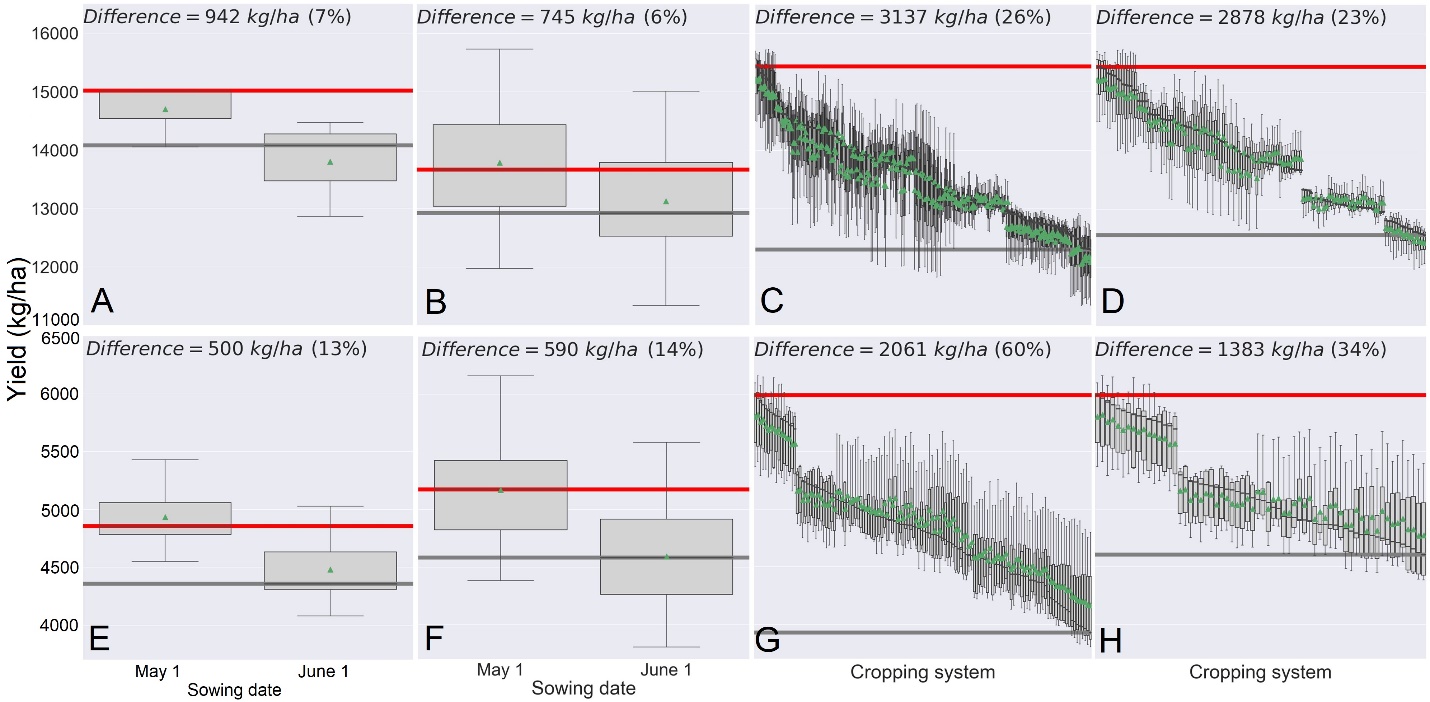


**Figure S9.** Simulated yield differences due to sowing date when using algorithms developed with 30-d weather variables (variables in **Table S8** and **S9** for maize and soybean, respectively). Maize yield difference (in kg/ha and percentage) due to sowing date (May 1^st^ vs June 1^st^) for a single identical background cropping system (A), maize yield difference due to sowing date when averaged across 256 (3 years × 256 cropping systems=768 year-specific yields) (B), maize yield variability in each of the 256 cropping systems (C), and maize yield variability in each of the 128 cropping systems with early sowing (D). Soybean yield difference due to sowing date (May 1^st^ vs June 1^st^) for a single identical background cropping system (E), soybean yield difference due to sowing date when averaged across 128 (5 years × 128 cropping systems=640 year-specific yields) (F), soybean yield in each of the 128 cropping systems (G) and soybean yield variability due in each of the 64 cropping systems with early sowing (H). Within each panel, the horizontal red and grey lines indicate the boxplot with maximum and minimum yield, respectively. In the left four panels, boxes delimit first and third quartiles; solid lines inside boxes indicate median and green triangles indicate means. Upper and lower whiskers extend to maximum and minimum yields. Each maize and soybean cropping system is a respective 8-way and a 7-way interaction of management practices in a randomly chosen field in Wisconsin, USA (**Table S3** and **S5**, respectively).

| **Category†** | **Variable** | **Percent missing data** |
| --- | --- | --- |
| E | RH pre (average relative humidity between 91-150 days of year) | 0 |
| E | Prcp pre (cumulative precipitation between 91-150 days of year) | 0 |
| E | Prcp season (cumulative precipitation between 151-270 days of year) | 0 |
| E | Prcp post (cumulative precipitation between 271-330 days of year) | 0 |
| E | Sumrad pre (cumulative solar radiation between 91-150 days of year) | 0 |
| E | Sumrad season (cumulative solar radiation between 151-270 days of year) | 0 |
| E | Vpd season (average vapor pressure deficit between 151-270 days of year) | 0 |
| E | Latitude (degrees) | 0 |
| E | Longitude (degrees) | 0 |
| E | Year | 0 |
| M | Sowing date (day of year) | 6 |
| G | Cultivar relative maturity (company rating) | 7.1 |
| M | Irrigation (yes/no) | 14.3 |
| E | Soil type (clay, silt, loam, sand, clay loam, loamy sand, sandy loam, silty clay, silty loam, sandy clay loam, silty clay loam) | 15 |
| M | Row spacing (cm) | 20 |
| G | Black cutworm trait (yes/no) | 22 |
| G | Corn earworm trait (yes/no) | 22 |
| G | Corn rootworm trait (yes/no) | 22 |
| G | Non-GMO (yes/no) | 22 |
| G | Drought tolerant trait (yes/no) | 22 |
| G | European corn borer trait (yes/no | 22 |
| G | Fall armyworm trait (yes/no) | 22 |
| G | Glufosinate resistant trait (yes/no) | 22 |
| G | Glyphosate resistant trait (yes/no) | 22 |
| G | Stalk borer trait (yes/no) | 22 |
| G | Sugarcane borer trait (yes/no) | 22 |
| G | Southwestern corn borer trait (yes/no) | 22 |
| G | True armyworm trait (yes/no) | 22 |
| G | Western bean cutworm trait (yes/no) | 22 |
| M | Nitrogen fertilizer (kg/ha) | 28.2 |
| M | Seeding rate (seeds/ha) | 27.9 |
| M | Use of manure (yes/no) | 28.6 |
| M | Potassium fertilizer (kg/ha) | 28.8 |
| M | Phosphorous fertilizer (kg/ha) | 29.1 |
| M | Previous crop (maize, soybean, cereal other than maize, legume other than soybean, cotton, fallow) | 33.7 |
| G | Insect target (none, above ground, below ground, both) | 35.3 |
| M | Tillage practices (conventional, reduced, no-till) | 43.6 |
| M | Biological seed treatment (yes/no) | 47.2 |
| M | Fungicide seed treatment (yes/no) | 47.2 |
| M | Insecticide seed treatment (yes/no) | 47.2 |
| M | Inoculant seed treatment (yes/no) | 47.2 |
| M | Nematicide seed treatment (yes/no) | 47.2 |

**Table S1**. Variables used in the maize algorithm along with percentage of missing observations. Variables are ranked in ascending percent of missing data order.

†Macro-categories of independent variables. M: management, G: genetics, E: environment

| **Category†** | **Variable** | **Percent missing data** |
| --- | --- | --- |
| E | RH pre (average relative humidity between 91-150 days of year) | 0 |
| E | RH post (average relative humidity between 271-330 days of year) | 0 |
| E | Prcp pre (cumulative precipitation between 91-150 days of year) | 0 |
| E | Prcp season (cumulative precipitation between 151-270 days of year) | 0 |
| E | Prcp post (cumulative precipitation between 271-330 days of year) | 0 |
| E | Sumrad pre (cumulative solar radiation between 91-150 days of year) | 0 |
| E | Sumrad season (cumulative solar radiation between 151-270 days of year) | 0 |
| E | Vpd season (average vapor pressure deficit between 151-270 days of year) | 0 |
| E | Latitude (degrees) | 0 |
| E | Longitude (degrees) | 0 |
| E | Year | 0 |
| G | Cultivar maturity group | 0.6 |
| G | Non-GMO (yes/no) | 4.9 |
| G | Glufosinate resistant trait (yes/no) | 4.9 |
| G | Glyphosate resistant trait (yes/no) | 4.9 |
| G | Xtend trait (yes/no) | 4.9 |
| G | Glyphosate 2 resistant trait (yes/no) | 4.9 |
| M | Irrigation (yes/no) | 5.7 |
| M | Sowing date (day of year) | 10.5 |
| M | Row spacing (cm) | 22.2 |
| M | Seeding rate (seeds/ha) | 28.4 |
| E | Soil type (clay, loam, sand, clay loam, loamy sand, sandy loam, silty clay, silty loam, silty clay loam) | 38.6 |
| M | Use of foliar fungicide (yes/no) | 54.2 |
| M | Tillage practices (conventional, reduced, no-till) | 57.3 |
| M | Previous crop (maize, rice, soybean, cereal other than maize and rice, legume other than soybean, cotton, fallow, tobacco) | 64.6 |

**Table S2**. Variables used in the soybean algorithm along with percentage of missing observations. Variables are ranked in ascending percent of missing data order.

†Macro-categories of independent variables. M: management, G: genetics, E: environment

| **Variable** | **Levels used** |
| --- | --- |
| Sowing date | May 1^st^, June 1^st^ |
| Tillage practice | Conventional, No-till |
| Seeding rate (seeds/ha) | 70,000, 90,000 |
| Nitrogen fertilizer (kg/ha) | 140, 220 |
| Phosphorous fertilizer (kg/ha) | 0, 40 |
| Cultivar relative maturity (company rating) | 100, 110 |
| Manure | yes, no |
| Previous crop | maize, soybean |

**Table S3**. Levels of variables used to generate the hypothetical cropping systems for maize. Each cropping system is a unique combination of the levels in the table holding constant the rest background management practices listed in **Table S1.**

|  | Highest yielding systems | Lowest yielding systems |
| --- | --- | --- |
| Nitrogen (kg/ha) | 220 | 140 |
| Phosphorous (kg/ha) | 40 | 0 |
| Maturity | 110 | 100 |
| Seeding rate (seeds/ha) | 90,000 | 70,000 |
| Previous crop | Soybean | Maize |
| Tillage practice | Conventional | No-till |
| Manure use | yes | no |

**Table S4**. Levels/rates of management practices in the 5% highest and lowest yielding maize cropping systems with early sowing date (May 1^st^).

| **Variable** | **Levels used** |
| --- | --- |
| Sowing date | May 1^st^, June 1^st^ |
| Tillage practice | Conventional, No-till |
| Seeding rate (seeds/ha) | 345,000, 400,000 |
| Row spacing (cm) | 35, 75 |
| Foliar fungicide use | yes, no |
| Cultivar maturity group | 1, 2 |
| Previous crop | maize, soybean |

**Table S5**. Levels of variables used to generate the hypothetical cropping systems for soybean. Each cropping system is a unique combination of the levels in the table holding constant the rest background management practices listed in **Table S2**.

|  | Highest yielding systems | Lowest yielding systems |
| --- | --- | --- |
| Cultivar maturity group | 2 | 1 |
| Seeding rate (seeds/ha) | 400,000 | 345,000 |
| Row spacing (cm) | 38 | 75 |
| Foliar Fungicide use | yes | no |
| Tillage practice | No-till | No-till |
| Previous crop | Maize | Soybean |

**Table S6**. Levels/rates of management practices in the 5% highest and lowest yielding soybean cropping systems with early sowing date (May 1^st^).

| **Weather parameter** | **Abbreviation in Figures S1 and S2 and Tables S1 and S2** | **Units** | **Description** |
| --- | --- | --- | --- |
| Day length | Dlength | minutes/day | Duration of the daylight period in minutes per day. |
| Precipitation | prcp | mm/day | Daily total precipitation in millimeters per day, sum of all forms converted to water equivalent. |
| Daily total radiation | sumrad | MJ/m2/day | Daily total radiation |
| Maximum air temperature | tmax | degrees ^o^C | Daily maximum 2-meter air temperature in degrees Celsius. |
| Minimum air temperature | tmin | degrees ^o^C | Daily minimum 2-meter air temperature in degrees Celsius. |
| Water vapor pressure deficit | vpd | kPa | Estimated as the difference between saturated vapor pressure (0.6107 * exp(17.269 × T/(237.3 + T))) at daily Tmax and at daily Tmin |
| Relative humidity | RH | % | Calculated by dividing the vapor pressure by the average of saturated vapor pressure at daily Tmax and Tmin. |
| Growing degree days | gdd | unitless | Calculated using 10 and 30 ^o^C as Tbase and Topt, respectively |

**Table S7**. Weather variables considered in the algorithms for both crops.

| **Variable** | **Percent missing data** |
| --- | --- |
| RH1 (average relative humidity between 1-30 days of year) | 0 |
| RH2 (average relative humidity between 31-60 days of year) | 0 |
| RH4 (average relative humidity between 91-120 days of year) | 0 |
| RH8 (average relative humidity between 211-240 days of year) | 0 |
| RH9 (average relative humidity between 241-270 days of year) | 0 |
| RH10 (average relative humidity between 271-300 days of year) | 0 |
| Prcp1 to 3 (cumulative precipitation between 1-90 days of year) | 0 |
| Prcp4 (cumulative precipitation between 91-120 days of year) | 0 |
| Prcp5 (cumulative precipitation between 121-150 days of year) | 0 |
| Prcp6 (cumulative precipitation between 151-180 days of year) | 0 |
| Prcp7 (cumulative precipitation between 181-210 days of year) | 0 |
| Prcp8 (cumulative precipitation between 211-240 days of year) | 0 |
| Prcp9 (cumulative precipitation between 241-270 days of year) | 0 |
| Prcp10 (cumulative precipitation between 271-300 days of year) | 0 |
| Prcp11 (cumulative precipitation between 301-330 days of year) | 0 |
| Sumrad2 (cumulative solar radiation between 31-60 days of year) | 0 |
| Sumrad3 (cumulative solar radiation between 61-90 days of year) | 0 |
| Sumrad4 (cumulative solar radiation between 91-120 days of year) | 0 |
| Sumrad5 (cumulative solar radiation between 121-150 days of year) | 0 |
| Sumrad6 (cumulative solar radiation between 151-180 days of year) | 0 |
| Sumrad7 (cumulative solar radiation between 181-210 days of year) | 0 |
| Sumrad8 (cumulative solar radiation between 211-240 days of year) | 0 |
| Vpd5 (average vapor pressure deficit between 121-150 days of year) | 0 |
| Vpd6 (average vapor pressure deficit between 181-210 days of year) | 0 |
| Vpd8 (average vapor pressure deficit between 271-300 days of year) | 0 |
| Latitude (degrees) | 0 |
| Longitude (degrees) | 0 |
| Year | 0 |
| Sowing date (day of year) | 6 |
| Cultivar relative maturity (company rating) | 7.1 |
| Irrigation (yes/no) | 14.3 |
| Soil type (clay, silt, loam, sand, clay loam, loamy sand, sandy loam, silty clay, silty loam, sandy clay loam, silty clay loam) | 15 |
| Row spacing (cm) | 20 |
| Black cutworm trait (yes/no) | 22 |
| Corn earworm trait (yes/no) | 22 |
| Corn rootworm trait (yes/no) | 22 |
| Non-GMO (yes/no) | 22 |
| Drought tolerant trait (yes/no) | 22 |
| European corn borer trait (yes/no | 22 |
| Fall armyworm trait (yes/no) | 22 |
| Glufosinate resistant trait (yes/no) | 22 |
| Glyphosate resistant trait (yes/no) | 22 |
| Stalk borer trait (yes/no) | 22 |
| Sugarcane borer trait (yes/no) | 22 |
| Southwestern corn borer trait (yes/no) | 22 |
| True armyworm trait (yes/no) | 22 |
| Western bean cutworm trait (yes/no) | 22 |
| Nitrogen fertilizer (kg/ha) | 27.1 |
| Seeding rate (seeds/ha) | 27.9 |
| Use of manure (yes/no) | 28.6 |
| Potassium fertilizer (kg/ha) | 28.8 |
| Phosphorous fertilizer (kg/ha) | 29.1 |
| Previous crop (maize, soybean, cereal other than maize, legume other than soybean, cotton, fallow) | 33.7 |
| Insect target (none, above ground, below ground, both) | 35.3 |
| Tillage practices (conventional, reduced, no-till) | 43.6 |
| Biological seed treatment (yes/no) | 47.2 |
| Fungicide seed treatment (yes/no) | 47.2 |
| Insecticide seed treatment (yes/no) | 47.2 |
| Inoculant seed treatment (yes/no) | 47.2 |
| Nematicide seed treatment (yes/no) | 47.2 |

**Table S8**. Variables used in the maize algorithm along with percentage of missing observations. Variables are ranked in ascending percent of missing data order. The difference from **Table S1** is the difference in weather variables only.

| **Variable** | **Percent missing data** |
| --- | --- |
| RH1 (average relative humidity between 1-30 days of year) | 0 |
| RH2 (average relative humidity between 31-60 days of year) | 0 |
| RH5 (average relative humidity between 121-150 days of year) | 0 |
| RH8 (average relative humidity between 211-240 days of year) | 0 |
| RH9 (average relative humidity between 241-270 days of year) | 0 |
| RH10 (average relative humidity between 271-300 days of year) | 0 |
| RH11 (average relative humidity between 301-330 days of year) | 0 |
| Prcp4 (cumulative precipitation between 91-120 days of year) | 0 |
| Prcp5 (cumulative precipitation between 121-150 days of year) | 0 |
| Prcp6 (cumulative precipitation between 151-180 days of year) | 0 |
| Prcp7 (cumulative precipitation between 181-210 days of year) | 0 |
| Prcp8 (cumulative precipitation between 211-240 days of year) | 0 |
| Prcp9 (cumulative precipitation between 241-270 days of year) | 0 |
| Prcp10 (cumulative precipitation between 271-300 days of year) | 0 |
| Prcp11 (cumulative precipitation between 301-330 days of year) | 0 |
| Sumrad2 (cumulative solar radiation between 31-60 days of year) | 0 |
| Sumrad3 (cumulative solar radiation between 61-90 days of year) | 0 |
| Sumrad4 (cumulative solar radiation between 91-120 days of year) | 0 |
| Sumrad5 (cumulative solar radiation between 121-150 days of year) | 0 |
| Sumrad6 (cumulative solar radiation between 151-180 days of year) | 0 |
| Sumrad7 (cumulative solar radiation between 181-210 days of year) | 0 |
| Sumrad8 (cumulative solar radiation between 211-240 days of year) | 0 |
| Sumrad9 (cumulative solar radiation between 241-270 days of year) | 0 |
| Vpd5 (average vapor pressure deficit between 121-150 days of year) | 0 |
| Vpd6 (average vapor pressure deficit between 151-180 days of year) | 0 |
| Vpd7 (average vapor pressure deficit between 181-210 days of year) | 0 |
| Vpd8 (average vapor pressure deficit between 211-240 days of year) | 0 |
| Latitude (degrees) | 0 |
| Longitude (degrees) | 0 |
| Year | 0 |
| Cultivar maturity group | 0.6 |
| Non-GMO (yes/no) | 4.9 |
| Glufosinate resistant trait (yes/no) | 4.9 |
| Glyphosate resistant trait (yes/no) | 4.9 |
| Xtend trait (yes/no) | 4.9 |
| Glyphosate 2 resistant trait (yes/no) | 4.9 |
| Irrigation (yes/no) | 5.7 |
| Sowing date (day of year) | 10.5 |
| Row spacing (cm) | 22.2 |
| Seeding rate (seeds/ha) | 28.4 |
| Soil type (clay, loam, sand, clay loam, loamy sand, sandy loam, silty clay, silty loam, silty clay loam) | 38.6 |
| Use of foliar fungicide (yes/no) | 54.2 |
| Tillage practices (conventional, reduced, no-till) | 57.3 |
| Previous crop (maize, rice, soybean, cereal other than maize and rice, legume other than soybean, cotton, fallow, tobacco) | 64.6 |

**Table S9**. Variables used in the soybean algorithm along with percentage of missing observations. Variables are ranked in ascending percent of missing data order. The difference from **Table S2** is the difference in weather variables only.

| **State** | **Website** |
| --- | --- |
| Alabama | https://aaes.auburn.edu/blog/category/variety-tests/ |
| Arkansas | https://aaes.uada.edu/variety-testing/ |
| Colorado | https://csucrops.agsci.colostate.edu/ |
| Delaware | https://www.udel.edu/academics/colleges/canr/cooperative-extension/sustainable-production/variety-trials/ |
| Florida | https://edis.ifas.ufl.edu/entity/topic/field_crop_variety_trials |
| Georgia | https://swvt.uga.edu/ |
| Illinois | http://vt.cropsci.illinois.edu/ |
| Indiana | https://extension.purdue.edu/jay/article/12861 |
| Iowa | http://www.croptesting.iastate.edu/ |
| Kansas | https://www.agronomy.k-state.edu/services/crop-performance-tests/ |
| Kentucky | https://graincrops.ca.uky.edu/variety-testing |
| Michigan | https://varietytrials.msu.edu/ |
| Minnesota | https://www.maes.umn.edu/publications/field-crop-trials/corn |
| Mississippi | https://www.mafes.msstate.edu/variety-trials/index.asp |
| Missouri | https://varietytesting.missouri.edu/ |
| Nebraska | https://cropwatch.unl.edu/varietytest |
| New York | https://cals.cornell.edu/school-integrative-plant-science/school-sections/plant-breeding-genetics-section/outreach-extension-plant-breeding-genetics |
| North Carolina | https://officialvarietytesting.ces.ncsu.edu/ |
| North Dakota | https://www.ag.ndsu.edu/varietytrials |
| Ohio | https://u.osu.edu/perf/ |
| Pennsylvania | https://extension.psu.edu/forage-and-food-crops/agronomic-crops/species-and-varieties |
| South Carolina | https://www.clemson.edu/cafls/research/vt/ |
| South Dakota | https://extension.sdstate.edu/agriculture/crops |
| Tennessee | https://search.utcrops.com/ |
| Texas | https://varietytesting.tamu.edu/ |
| Vermont | https://www.uvm.edu/extension/nwcrops/research |
| Virginia | http://www.virginiacrop.org/virginia-tech-variety-testing.html |
| Wisconsin | https://fyi.extension.wisc.edu/fieldcroppathology/university-of-wisconsin-variety-trials/ |

**Table S10**. Websites hosting all state-specific variety trial data.
